# Qualitative Transcriptional Signature for the Pathological Diagnosis of Pancreatic Cancer

**DOI:** 10.3389/fmolb.2020.569842

**Published:** 2020-09-23

**Authors:** Yu-Jie Zhou, Xiao-Fan Lu, Jia-Lin Meng, Xin-Yuan Wang, Xin-Jia Ruan, Chang-Jie Yang, Qi-Wen Wang, Hui-Min Chen, Yun-Jie Gao, Fang-Rong Yan, Xiao-Bo Li

**Affiliations:** ^1^Division of Gastroenterology and Hepatology, Key Laboratory of Gastroenterology and Hepatology, Ministry of Health, Shanghai Institute of Digestive Disease, Renji Hospital, School of Medicine, Shanghai Jiao Tong University, Shanghai, China; ^2^State Key Laboratory of Natural Medicines, Research Center of Biostatistics and Computational Pharmacy, China Pharmaceutical University, Nanjing, China; ^3^Department of Urology, The First Affiliated Hospital of Anhui Medical University, Hefei, China; ^4^Department of Liver Surgery, Renji Hospital, School of Medicine, Shanghai Jiao Tong University, Shanghai, China

**Keywords:** molecular signature, relative expression orderings, early diagnosis, pancreatic cancer, gene pairs

## Abstract

It is currently difficult for pathologists to diagnose pancreatic cancer (PC) using biopsy specimens because samples may have been from an incorrect site or contain an insufficient amount of tissue. Thus, there is a need to develop a platform-independent molecular classifier that accurately distinguishes benign pancreatic lesions from PC. Here, we developed a robust qualitative messenger RNA signature based on within-sample relative expression orderings (REOs) of genes to discriminate both PC tissues and cancer-adjacent normal tissues from non-PC pancreatitis and healthy pancreatic tissues. A signature comprising 12 gene pairs and 17 genes was built in the training datasets and validated in microarray and RNA-sequencing datasets from biopsy samples and surgically resected samples. Analysis of 1,007 PC tissues and 257 non-tumor samples from nine databases indicated that the geometric mean of sensitivity and specificity was 96.7%, and the area under receiver operating characteristic curve was 0.978 (95% confidence interval, 0.947–0.994). For 20 specimens obtained from endoscopic biopsy, the signature had a diagnostic accuracy of 100%. The REO-based signature described here can aid in the molecular diagnosis of PC and may facilitate objective differentiation between benign and malignant pancreatic lesions.

## Introduction

Pancreatic cancer (PC) is the fourth leading cause of cancer deaths in the United States and the sixth leading cause in China. Patients with PC have a 5-year survival rate of 8.5% in the United States and 7.2% in China (Siegel et al., 2019; Zhao et al., 2019). The diagnosis and treatment of pancreatic cancer remain challenging. Patients with early-stage PC are usually asymptomatic, and only about 10% of patients are diagnosed at an early stage (Singhi et al., 2019). Serum cancer antigen 19-9 (CA 19-9) is the only marker approved by the United States Food and Drug Administration for use in the routine management of PC. Imaging techniques, such as computed tomography (CT), magnetic resonance imaging (MRI), endoscopic ultrasonography (EUS), and endoscopic retrograde cholangiopancreatography (ERCP), can help in the diagnosis of PC. EUS is currently the most effective imaging method for diagnosis and is superior to CT and MRI (Costache et al., 2017). However, the accuracy of EUS for early detection of PC is still unsatisfactory, and it is often difficult to distinguish benign and malignant pancreatic lesions (Singhi et al., 2019).

Therefore, it is sometimes necessary to perform a tissue biopsy for the pathological diagnosis of PC. In clinical practice, more accurate and definitive pathological diagnoses can be made using biopsies from EUS-guided fine needle aspiration (EUS-FNA), and this method has an overall diagnostic accuracy of 91% (Banafea et al., 2016). However, biopsy samples may be collected from incorrect locations, and this can lead to false-negative results. Repeated EUS-FNA not only increases the diagnostic accuracy to 96.3% but also increases the risk of complications (Suzuki et al., 2013). Thus, it is vital to develop molecular signatures to complement the present histological methods for diagnosis of early PC, especially when the locations of biopsy samples are incorrect. Recent studies reported that within-sample relative expression orderings (REOs) of genes are insensitive to experimental batch effects and can provide qualitative transcriptional signatures that can be applied to samples at an individual level (Eddy et al., 2010; Wang et al., 2015). Within-sample REOs are also insensitive to variable proportions of tumor epithelial cells sampled from different tumor locations in the same patient (Cheng et al., 2017), RNA degradation during specimen storage and preparation (Chen et al., 2017), and amplification bias for minimum specimens, which can lead to failure of quantitative transcriptional signatures in clinical applications (Liu et al., 2017). Thus, within−sample REOs may provide a robust qualitative signature for the early diagnosis of PC.

In this study, based on the REOs of 12 gene pairs, we identified a qualitative transcriptional signature for the early diagnosis of PC. The signature can accurately discriminate both PC tissues and adjacent-normal tissues from normal pancreatic tissues and non−PC pancreatitis in both biopsy and surgical resection samples.

## Materials and Methods

### Data and Preprocessing

Transcriptional profiles of pancreatic tissues were retrieved from Gene Expression Omnibus (GEO), the Genotype-Tissue Expression (GTEx) project, and the Cancer Genome Atlas (TCGA) Data Portal^1^ and were measured by the Illumina, Affymetrix, or Agilent platforms. The training cohort consisted of 5 microarray datasets (GSE101462, GSE71989, GSE91035, E-MEXP-1121, and E-MTAB-1791), with 74 normal pancreatic tissues, 72 pancreatitis tissues, and 269 PC samples (Table 1). The tuning dataset (GSE41368) consisted of 6 normal pancreas tissues and 6 PC tissues, and the external validation cohort consisted of 9 microarray and RNA-sequencing datasets of 258 normal pancreatic samples and 1,051 tumor-related pancreatic lesions (GSE50827, GSE19650, GSE62165, GSE43288, GSE21501, GSE71729, E-MTAB-6134, GTEx, and TCGA).

**TABLE 1:** Datasets used in this study.

Dataset	Sampling method	Platform	Normal pancreas	Pancreatitis	Precursor lesions	Pancreatic cancer	Adjacent normal

Datasets used for identification of the qualitative signature							
GSE101462	FF or FFPE tissues from surgery	Illumina GPL10558	10 FFPE	3 FF + 1 FFPE	–	3 FF + 3 FFPE	–
GSE71989	FF tissues from surgery	Affymetrix GPL570	–	–	–	14	–
GSE91035	FF tissues from surgery	Agilent GPL22763	8	–	–	27	–
E-MEXP-1121	Microdissected tissue samples	Affymetrix GPL96	–	9 chronic pancreatitis	–	27	–
E-MTAB-1791	FF tissues from surgery	Illumina human WG6 BeadChip v3	56	59	–	195	–
GSE41368	FF tissues from surgery	Affymetrix Human Gene 1.0 ST Array	6	–	–	6	–

**Datasets used for evaluating the performance of the qualitative signature**							

GSE50827	FF tissues from surgery	Illumina GPL10558	–	–	–	103	–
GSE19650	Microdissected tissue samples	Affymetrix GPL570	7	–	3 IPMNs + 6 IPMAs	6 IPMCs	–
GSE62165	FF tissues from surgery	Affymetrix GPL13667	–	–	–	118	13
GSE43288	Biopsy	Affymetrix GPL96	3	–	13 PanIN-2/3	4	–
GSE21501	FF tissues from surgery	Agilent GPL4133	–	–	–	102	–
GSE71729	FF tissues from surgery	Agilent GPL20769	–	–	–	145	46
E-MTAB-6134	FF tissues from surgery	Affymetrix GPL13667	–	–	–	309	–
GTEx		Illumina TrueSeq RNA sequencing	248	–	–	–	–
TCGA	FF tissues from surgery	Illumina HiSeq_RNASeqV2	–	–	–	179	4
Total			338	72	22	1,241	63

**GSE, accession numbers of datasets in Gene Expression Omnibus; FF, fresh-frozen; FFPE, formalin-fixed, paraffin-embedded; IPMN, intraductal papillary mucinous neoplasm; IPMA, intraductal papillary mucinous adenoma; IPMC, intraductal papillary mucinous carcinoma; PanIN, pancreatic intraepithelial neoplasia; GTEx, the Genotype-Tissue Expression project; TCGA, The Cancer Genome Atlas.**

Transcriptome HTSeq-counts data of the TCGA-Pancreatic Adenocarcinoma (PAAD) project were downloaded from the Genomic Data Commons using the R package “*TCGAbiolink*,” including 183 non-formalin-fixed and paraffin-embedded (FFPE) samples of primary pancreatic tumors. Ensembl ID for protein-coding messenger RNAs (mRNAs) was annotated to symbol name using GENCODE27. The number of fragments per kilobase of non-overlapped exons per million fragments mapped (FPKM) was calculated first and was then transformed into transcripts per kilobase million (TPM) values. All mRNAs with TPM values < 1 in more than 90% of the samples were considered to be noise and removed prior to downstream analysis. Data of the GTEx project were retrieved from the UCSC Xena browser^2^. For microarray datasets measured by Affymetrix platforms, the robust multiarray average (RMA) procedure was performed, with raw CEL files for background, using the R package “*affy*” for adjustment (Irizarry et al., 2003). For other platforms, the processed data were obtained from GEO and utilized for subsequent analyses.

### Identification of Qualitative REO-Based PC Diagnosis Signature

For the purpose of this study, tumor samples were identified as “cancer” or “cancer-adjacent normal” because the transcriptional characteristics of apparently normal tissue that is adjacent to a tumor differs from healthy normal tissues (Aran et al., 2017), whereas non-tumor samples involve “healthy normal” or “pancreatitis.”

**FIGURE 1:**
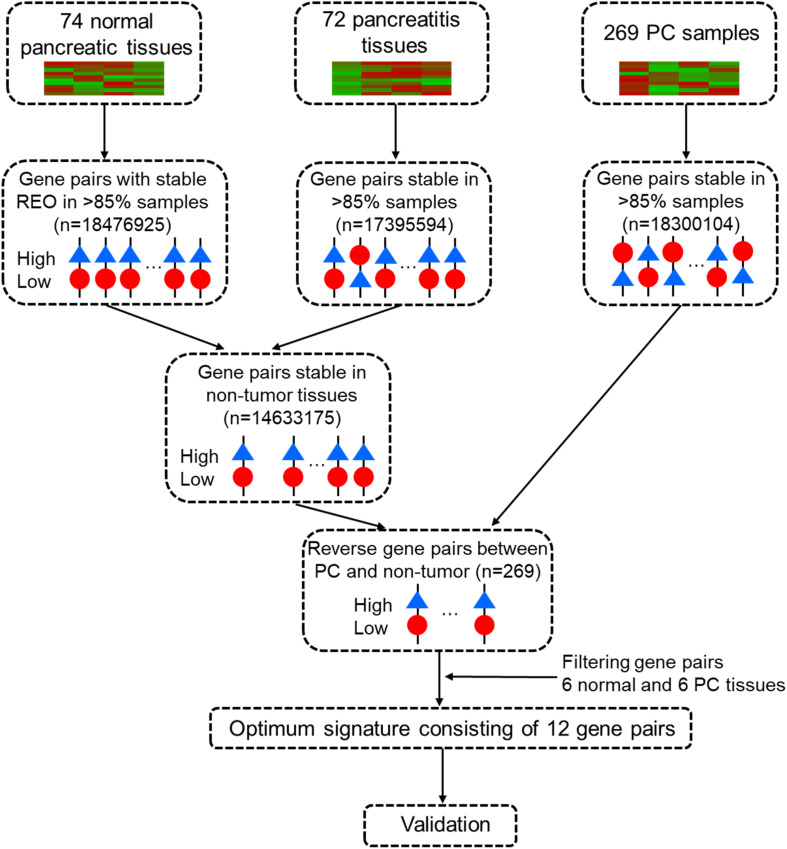
Workflow for identification and evaluation of a qualitative diagnostic signature of pancreatic cancer based on relative expression orderings (REOs).

Data analysis consisted of several sequential steps (Figure 1). First, a pairwise gene or REO within a sample with genes *i* and *j* was assigned a value of “1” if gene “i” had higher expression and “0” if gene j had greater expression. A “reversal gene pair” (RGP) was defined by the presence of the same REO pattern in more than 85% of the tumor samples (“cancer” and “cancer-adjacent normal”) and a reversed pattern in more than 85% of the non-tumor samples (“healthy normal” and “pancreatitis”) in the training dataset. Then, RGPs were filtered using the tuning dataset to establish a candidate REO signature of PC. The rank difference for each RGP was computed for each sample as:

R=i⁢jR-iRj

where *R*_*i*_ and *R*_*j*_ represent the rank of gene *i* and *j* within a sample, and *R*_*ij*_ represents the rank difference.

Then, *R*_*ij*_, the geometric mean of the mean value of *R*_*ij*_ in tumor samples was used to assess the extent of reversal of the gene pair between tumor and non-tumor samples:

$$\bar{R_{i\,j}}=\sqrt{\bar{\left|\bar{R_{i\,j}^{T}}\right|\times \left|\bar{R_{i\,j}^{N}}\right|}}$$

where $\bar{R_{i\,j}^{T}}$ is the mean value in tumor samples, and $\bar{R_{i\,j}^{N}}$ is the mean value in non-tumor samples. Ideally, the sign for each $R_{i\,j}^{X}$ should be uniform in each sample type, *X*{*X* ∈ (*T*,*N*)}. However, in most cases, a sample in a specific *X* may have an $R_{i\,j}^{X}$ with a different sign, and this can cause bias because of the absolute value operation. Therefore, an $R_{i\,j}^{X}$ with the “wrong” sign was forced to zero before subsequent analysis. Thus, a higher $\bar{R_{i\,j}}$ value corresponds to a larger reversal of the REO of the gene pair between tumor and non-tumor samples.

The selected candidate REO signatures were then sorted in a descending order according to their $\bar{R_{i\,j}}$ values, and the RGP with the largest $\bar{R_{i\,j}}$ was set as the seed. Then, a forward selection procedure was used, with one RGP entered at a time, to evaluate the classification accuracy based on a voting rule. Thus, if more than half of the RGPs of a sample in a signature framework had an REO for the tumor, the sample was classified as “tumor”; otherwise, it was classified as “non-tumor”. This selection procedure eventually selected the minimum and optimal number of RGPs with the highest accuracy.

### Performance Evaluation

All samples in the training, tuning, and validation datasets were first pooled together to assess the general predictive performance in different samples (tumor, cancer-adjacent normal, pancreatitis, and healthy normal) using receiver operating characteristic (ROC) analysis and calculation of the area under the curve (AUC). The accuracy was defined as the portion of correctly identified samples in the entire cohort, and the AUC, sensitivity, and specificity were calculated using the R package “*pROC*”. The diagnostic performance was further evaluated in each independent dataset by calculation of sensitivity and specificity. In this procedure, sensitivity refers to the proportion correctly identified true positives and specificity to the proportion of correctly identified true negatives. Sensitivity and specificity were recorded at the median cutoff of the voting threshold.

### Statistical Analyses

All statistical analyses were performed using R software (version: 3.6.1^3^). The data analysis source code is provided in the github: https://github.com/xlucpu/PCsig.

## Results

### Identification of the Qualitative Gene Pair Signature for PC

We first used the training cohort from a merger of five microarray datasets (GSE101462, GSE71989, GSE91035, E-MEXP-1121, and E-MTAB-1791) to identify common gene pairs with stable REOs in 74 normal pancreatic tissues (18,476,925 gene pairs) and 72 pancreatitis samples (17,395,594 gene pairs). Then, we identified 14,633,175 gene pairs with identical REO patterns in at least 85% of normal pancreas and pancreatitis samples as stable gene pairs of non-tumor samples. We also identified 18,300,104 gene pairs with stable REO patterns in at least 85% of the 269 PC samples in the training cohort. Next, we used these data to identify 269 RGPs between the non-tumor and tumor tissues (Supplementary Table S1). After a tuning procedure using 6 normal pancreas and 6 PC tissues, we selected 20 gene pairs with identical REO patterns in the testing dataset (GSE41368).

**FIGURE 2:**
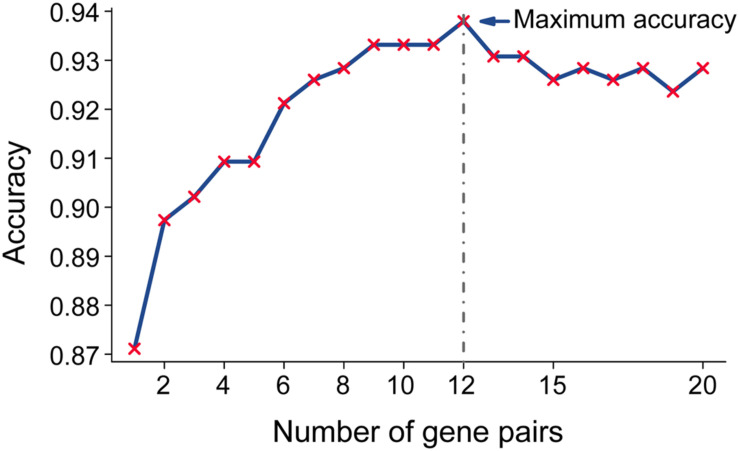
Accuracy of top-ranked gene pairs among the 20 reversal gene pairs (RGPs) in the training data. Twenty RGPs were sorted in a descending order according to the extent of reversal between tumor and non-tumor tissues in the training datasets. Twelve gene pairs provided the highest classification accuracy according to the “majority voting rule” and were used for the qualitative diagnostic signature.

Then, we sorted the 20 RGPs into descending order based on the rank difference ($\bar{R_{i\,j}}$) between PC and non-tumor tissues (normal pancreas and pancreatitis) in the merged data from the training set and utilized the top−ranked *k* gene pairs for sample classification using majority vote rule. The results indicated that for *k* ranging from 1 to 20, the largest geometric mean of sensitivity and specificity (93.79%) occurred for *k* = 12 (Figure 2). We thus identified these 12 gene pairs (Table 2) as the transcriptional signature for discriminating tumor and non-tumor samples.

**TABLE 2:** Twelve gene-pair signature and 17 genes used for early diagnosis of PC.

Signature	Gene A	Gene B
Pair 1	LAMC2	TEX11
Pair 2	LAMC2	HDAC11
Pair 3	PITX1	KCNH6
Pair 4	S100P	AP1M1
Pair 5	LAMC2	FOXRED2
Pair 6	S100P	AIP
Pair 7	LAMC2	MYOM2
Pair 8	CST6	VIPR2
Pair 9	CDH3	EXOSC5
Pair 10	S100P	MAP1LC3B
Pair 11	CST6	KIRREL2
Pair 12	CDH3	TP53RK

**Genes in column A had higher expression in PC tissues, and genes in column B had higher expression in non-PC samples. PC, pancreatic cancer; LAMC2, laminin γ2-chain; PITX1, paired like homeodomain 1; S100P, S100 calcium-binding protein P; CST6, cystatin 6; CDH3, cadherin-3; TEX11, testis-expressed protein 11; HDAC11, histone deacetylase 11; KCNH6, potassium voltage-gated channel subfamily H member 6; AP1M1, adaptor related protein complex 1 subunit mu 1; FOXRED2, FAD-dependent oxidoreductase domain containing 2; AIP, aryl hydrocarbon receptor interacting protein; MYOM2, myomesin 2; VIPR2, vasoactive intestinal peptide receptor 2; EXOSC5, exosome component 5; MAP1LC3B, microtubule associated protein 1 light chain 3β; KIRREL2, kirre-like nephrin family adhesion molecule 2; TP53RK, tumor protein 53 regulating kinase.**

### Validation of the Diagnostic Signature in External Validation Datasets

**FIGURE 3:**
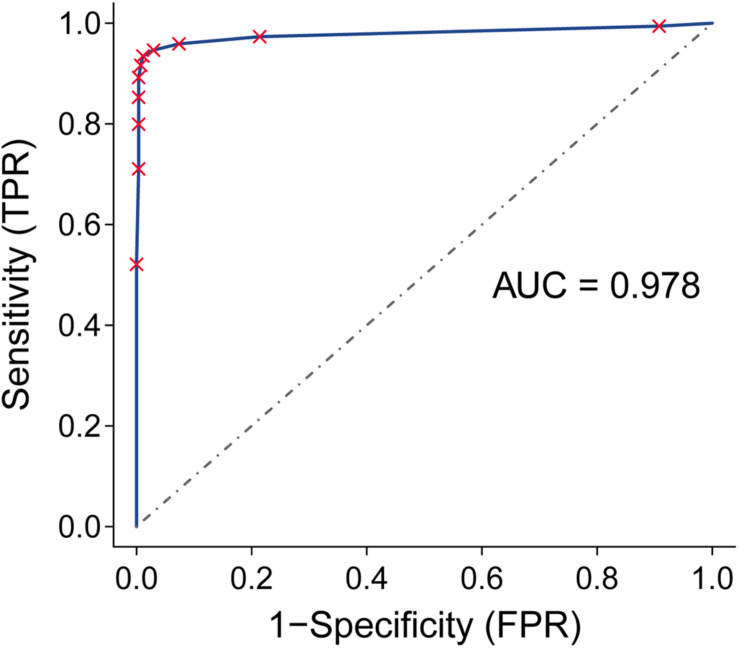
Receiver operating characteristic (ROC) analysis and area under the curve (AUC) for the qualitative diagnostic signature in nine validation datasets.

Next, we assessed the performance of the 12-gene pair signature to discriminate PC (including cancer-adjacent tissue) from non-tumor samples. For the 1,007 PC tissues and 257 non-tumor samples from the 9 external validation databases, the geometric mean of sensitivity and specificity was 96.7% and the area under receiver operating characteristic curve was 0.978 (95% confidence interval, 0.947–0.994; Figure 3).

Notably, our analysis of 17 PC and 3 normal samples obtained from endoscopic biopsies (GSE43288) had a diagnostic accuracy of 100%. For normal pancreatic tissues obtained by autopsy (GTEx), our method correctly classified all 248 samples as normal pancreatic tissue. The other validation sets consisted of samples from surgically resected tissues. For data measured by microarray, our signature correctly identified 96.07% of 842 tumor tissues as tumor samples. The details of diagnostic performance for the transcriptional signature are shown in Table 3 and Supplementary Table S2.

**TABLE 3:** Performance of the gene signature in the validation datasets (surgically resected samples and biopsy samples).

Dataset	Sampling method	Number (sensitivity) of tumor samples	Number (specificity) of non-tumor tissues
GSE50827	Surgery	98 (95.1%)	−
GSE19650	Surgery	15 (100%)	6 (85.8%)
GSE62165	Surgery	121 (92.3%)	−
GSE43288	Biopsy	17 (100%)	3 (100%)
GSE21501	Surgery	97 (94.7%)	−
GSE71729	Surgery	182 (95.3%)	−
E-MTAB-6134	Surgery	306 (99%)	−
GTEx	−	−	248 (100%)
TCGA	Surgery	171 (93.4%)	−

## Discussion

PC is expected to become the leading cause of cancer-specific mortality in Western countries by 2030, and yet early diagnosis remains difficult (Rahib et al., 2014). It is therefore necessary to identify a molecular diagnostic signature to reduce the uncertainty of pathological diagnosis due to sample error. In this study, we developed and validated a qualitative REO-based signature consisting of 12 different gene pairs with 17 genes for the early and accurate molecular diagnosis of PC. The proposed transcriptional signature can discriminate malignant tissues and most PC-adjacent tissues from benign tissues. Because the signature is effective even when sample site was inaccurate due to imperfect biopsy procedures, this can help to prevent the need for a second procedure.

In contrast to previous studies that used a transcriptomic diagnostic signature based on complicated procedures of data normalization and parameter fitting (Klett et al., 2018; Long et al., 2019), we analyzed the relative expression of gene pairs, instead of the expression of single genes, to differentiate PC from benign lesions. Our transcriptional REO-based signature employed relative ranking of gene expression by identifying multiple gene pairs and can be used without confounding from the batch effect and use of different sequencing platforms (Eddy et al., 2010). Previous studies successfully used this approach in the molecular diagnosis of colorectal cancer, gastric cancer, and hepatocellular carcinoma (Ao et al., 2018; Guan et al., 2019; Yan et al., 2019). The recent developments of high-throughput sequencing technologies have accompanied dramatic decreases in price. Given the limited amount of tissue sampled from biopsies, it is more efficient to measure the expression of a set of genes as markers for aiding pathological diagnosis, molecular subtype classification (Danilova et al., 2019), and chemoresistance of PC as part of the approach of “whole genome sequencing for all” (Xuan et al., 2013; Shao et al., 2018).

Several genes in the transcriptional signature proposed here have established roles in the carcinogenesis of PC. For instance, laminin γ2-chain (LAMC2) is a well-known PC-related gene whose level is elevated in the circulation of PC patients (Katayama et al., 2005). This gene upregulates mesenchymal markers in the microenvironment by activating the Akt/NHE1 signaling pathway and thus mediates the invasion and metastasis of cancer cells (Wang et al., 2020). Cystatin 6 (CST6) is overexpressed in pancreatic ductal adenocarcinoma (PDAC) cells and can stimulate PDAC cell growth by reducing the activity of intracellular cathepsin B (Hosokawa et al., 2008). There is evidence that S100 calcium-binding protein P (S100P) can be used as a biomarker in duodenal fluid and fine needle aspiration biopsies for detection of PDAC, and this protein has a diagnostic sensitivity of 84.8% in biopsy samples (Matsunaga et al., 2017; Aksoy-Altinboga et al., 2018). S100P secretes matrix metalloproteinase 9 (MMP9) and regulates the invasion of PC cells into the lymphatic endothelial monolayer, thereby promoting tumor cell invasion and metastasis (Dakhel et al., 2014; Nakayama et al., 2019). Cadherin-3 (CDH3) regulates cell migration and tumor growth by interacting with cadherin-1 in PC and is upregulated during early-stage PC (Siret et al., 2018).

There were some limitations in our study. First, due to the limited number of biopsy tissues, we only used samples collected from surgery (not from biopsy) in the training set, and this may have led to selection bias. Second, our study design was retrospective, with genomic data derived from publicly available databases. Prospective clinical studies are needed to validate our findings.

In summary, we constructed and validated an REO-based signature consisting of 12 gene pairs and 17 genes that can aid in the early diagnosis of PC.

## Data Availability Statement

All datasets generated for this study are included in the article/Supplementary Material.

## Ethics Statement

Ethical review and approval was not required for the study on human participants in accordance with the local legislation and institutional requirements. The patients/participants provided their written informed consent to participate in this study.

## Author Contributions

Y-JZ and C-JY obtained data from TCGA, GEO, and GTEx database, designed the study, and wrote the manuscript. Y-JZ, X-FL, J-LM, X-YW, Q-WW, Y-JG, and H-MC analyzed and interpreted the data. X-FL was responsible for the statistical analyses. X-BL and F-RY contributed to conception, design, and funding. All authors have been involved in revising and proofreading of the manuscript, and approved the manuscript.

## Conflict of Interest

The authors declare that the research was conducted in the absence of any commercial or financial relationships that could be construed as a potential conflict of interest.

## Footnotes

1 https://tcga-data.nci.nih.gov/tcga/

2 https://xenabrowser.net/datapages/

3 http://www.r-project.org/
