# Supplementary material for: Qualitative Transcriptional Signature for the Pathological Diagnosis of Pancreatic Cancer
Source: Front Mol Biosci. 2020 Sep 23;7:569842. doi: 10.3389/fmolb.2020.569842 (PMC7538791; doi:10.3389/fmolb.2020.569842)
Supplement: Supplementary file 1 [file Table_1.DOCX]

**Table S1.** Number of stable and reversal gene pairs in the training data.

| Sample type | Number of stable gene pairs | Number of overlap stable gene pairs | Number of reversal gene pairs |
| --- | --- | --- | --- |
| Normal | 18476925 | 14633175 | 20 |
| Pancreatitis | 17395594 |  |  |
| Pancreatic cancer | 18300104 | - |  |
