# Supplementary material for: Qualitative Transcriptional Signature for the Pathological Diagnosis of Pancreatic Cancer
Source: Front Mol Biosci. 2020 Sep 23;7:569842. doi: 10.3389/fmolb.2020.569842 (PMC7538791; doi:10.3389/fmolb.2020.569842)
Supplement: Supplementary file 2 [file Table_2.docx]

**Table S2.** Median fold change (FC) of each signature gene pair in different datasets of tumor samples (“cancer” and “cancer-adjacent normal”) and non-tumor samples (“normal pancreas” and “pancreatitis”) in the validation cohort.

| Dataset | Tumor samples group | | | | | | | | | | | |
| --- | --- | --- | --- | --- | --- | --- | --- | --- | --- | --- | --- | --- |
|  | Gene pair1 | Gene pair2 | Gene pair3 | Gene pair4 | Gene pair5 | Gene pair6 | Gene pair7 | Gene pair8 | Gene pair9 | Gene pair10 | Gene pair11 | Gene pair12 |
| GSE50827 | 1.66 | 1.38 | 1.59 | 1.28 | 1.47 | 1.35 | 1.42 | 1.55 | 1.26 | 1.29 | 1.88 | 1.21 |
| GSE19650 | 25.32 | 8.63 | 2.09 | 342.17 | 37.68 | 95.36 | 8.38 | 2.36 | 0.73 | 52.31 | 3.68 | 1.16 |
| GSE62165 | 1.82 | 1.97 | 1.87 | 2.34 | 1.49 | 1.75 | 1.59 | 2 | 1.26 | 1.39 | 2 | 1.33 |
| GSE43288 | 1.06 | 1.02 | 1.05 |  | 1.22 | 1.43 | 1.3 | 1.04 | 1.1 | 1.29 |  | 1.26 |
| GSE21501 | 2.29 | 31.9 | -0.49 | -2.54 | -3.28 | 12.95 | 7.84 | 15.06 | -3.19 | -19.73 | 7.76 | -14.71 |
| GSE71729 | 3.15 | 2.73 | 1.18 | 2.38 | 3.32 | 1.57 | 2.11 | 2.56 | 2 | 1.82 | 2.15 | 2.16 |
| E-MTAB-6134 | 2.37 | 1.45 | 1.78 | 1.54 | 1.65 | 1.3 | 1.72 | 1.39 | 1.25 | 1.19 | 1.55 | 1.65 |
| GTEx |  |  |  |  |  |  |  |  |  |  |  |  |
| TCGA | 227.66 | 17.93 | 3.69 | 61.26 | 44.66 | 5.55 | 277.52 | 81.99 | 1.55 | 11.76 | 9.49 | 3.1 |
|  |  |  |  |  |  |  |  |  |  |  |  |  |
|  |  |  |  |  |  |  |  |  |  |  |  |  |
| Dataset | Non-tumor samples group | | | | | | | | | | | |
|  | Gene pair1 | Gene pair2 | Gene pair3 | Gene pair4 | Gene pair5 | Gene pair6 | Gene pair7 | Gene pair8 | Gene pair9 | Gene pair10 | Gene pair11 | Gene pair12 |
| GSE50827 |  |  |  |  |  |  |  |  |  |  |  |  |
| GSE19650 | 0.03 | 1.04 | 1.58 | 4.42 | 5.41 | 1.08 | 0.41 | 0.45 | 0.22 | 0.85 | 0.42 | 0.87 |
| GSE62165 |  |  |  |  |  |  |  |  |  |  |  |  |
| GSE43288 | 0.69 | 0.69 | 0.99 |  | 0.9 | 0.79 | 0.91 | 0.88 | 0.76 | 0.81 |  | 0.96 |
| GSE21501 |  |  |  |  |  |  |  |  |  |  |  |  |
| GSE71729 |  |  |  |  |  |  |  |  |  |  |  |  |
| E-MTAB-6134 |  |  |  |  |  |  |  |  |  |  |  |  |
| GTEx | 0.31 | 0.17 | 0.34 | 0.37 | 1.02 | 0.03 | 4.06 | 0.09 | 0.03 | 0.07 | 0.01 | 0.12 |
| TCGA |  |  |  |  |  |  |  |  |  |  |  |  |

Gene pair1, gene pair2, …, gene pair12 represent LAMC2-TEX11, LAMC2-HDAC11, …, and CDH3-TP53RK, respectively, as indicated in Table 2.
